# The complete mitochondrial genome sequence of the giant mudskipper, *Periophthalmodon schlosseri* (Perciformes: gobiidae)

**DOI:** 10.1080/23802359.2016.1202742

**Published:** 2016-08-31

**Authors:** Yunhai Yi, Kai Zhang, Jieming Chen, Zhiqiang Ruan, Xinxin You, Qiong Shi

**Affiliations:** a BGI Education Center, University of Chinese Academy of Sciences, Shenzhen, China;; b Shenzhen Key Lab of Marine Genomics, Guangdong Provincial Key Lab of Molecular Breeding in Marine Economic Animals, BGI, Shenzhen, China;; c College of Life Sciences, Shenzhen University, Shenzhen, China

**Keywords:** Mitochondrial genome, *Periophthalmodon schlosseri*, phylogenetic tree

## Abstract

The complete mitochondrial genome sequence of the giant mudskipper, *Periophthalmodon schlosseri*, was first reported in this study. The circle genome is 16 470 bp in length, with the base composition of 28.4% T, 15.4% G, 29.2% A and 27.0% C. The mitogenome includes 13 protein-coding genes, 22 transfer RNA genes, two ribosomal RNA genes and one D-loop region. Only the NADH dehydrogenase subunit 6 (NAD6) and eight tRNA genes are encoded on the light strand. The mitochondrial gene arrangement of *P. schlosseri* is similar to those of most other gobies. The phylogenetic analysis using the neighbor-joining method showed that the kinship between *Periophthalmodon* and *Periophthalmus* is closer than those between *Periophthalmodon* and other selected genera. Our complete mitogenome data are going to provide the basis for taxonomic and phylogenetic research of amphibious mudskippers.

Mudskippers are very typical amphibious fishes living in mangrove area. Although there are just four genera, it is such a huge reservoir of valuable genes for exploration. The special living environment has endowed these species with amazing physiological and ecological adaptions. The genomes of four mudskippers represented four genera have revealed significant genetic changes in these amphibious species (You et al. 2014). Mitogenomic information is another important molecular tool for biodiversity protection. The mitogenomes of other three genera, including Scartelaos, Boleophthalmus and Periophthalmus, have been reported before (Liu et al. 2012; Qiu et al. 2015; Li et al. 2016). In this paper, the complete mitochondrial genome sequence of *Periophthalmodon schlosseri* (GenBank accession no. KX355324), a species from the left genus of mudskippers, was reported at the first time.

The fish sample was collected from Singapore (E103°51’, N1°16’) and stored in 95% ethanol. The voucher specimen is kept at China National Genebank (Accession no. SG2015051401). Total genomic DNA was extracted from the fins using traditional phenol–chloroform extraction method (Taggart et al. 1992). A library of the whole mitogenome with an insert size of 250 bp was constructed and sequenced by an Illumina HiSeq4000 platform (Illumina Inc., San Diego, CA) at BGI, Shenzhen, China. Raw reads were filtered with a Perl script that removed reads containing adaptor contamination. De novo assemblies were generated using SOAP denovo-Trans (-K 71) (Ruan et al. 2016).

The complete mitochondrial genome is 16 470 bp in length, with the base composition of 28.4% T, 15.4% G, 29.2% A and 27.0% C. The G + C content is 42.4% and the A + T ratio is 57.6%. The mitogenome includes 13 protein-coding genes, 22 tRNA genes and two rRNA genes (12S rRNA and 16S rRNA), annotated by DOGMA (http://dogma.ccbb.utexas.edu)(Wyman et al. 2004). The arrangement of all genes is identical to other gobies (Gan et al. 2015). All the protein-coding genes are initiated with ATG codon, except for COXI that starts with GTG. The length of the 22 tRNA genes varies from 65 to 75 bp. Most of the genes are encoded on the heavy strand, except for eight tRNA genes and the NAD6 gene encoded on the light strand. The 12S and 16S ribosomal RNA genes are located between the tRNAPhe and tRNALeu genes, and are separated by the tRNAVal gene. The D-loop control region, consisting of 815 nucleotides, is located between tRNAPro and tRNAPhe genes.

A phylogenetic tree was constructed by the neighbour-joining and maximum-likelihood methods using MEGA6.0 (Tamura et al. 2013) software with 15 more complete mitochondrial genome sequences downloaded from NCBI (http://www. ncbi.nlm.nih.gov/). Both the two methods generated the identical topological structure (Figure 1). The fishes in the suborder Gobioidei are grouped together while other fish species are formed side branches. *P. schlosseri* is clustered into one clade with other species from *Periophthalmus*, which is then grouped with *Boleophthalmus* and *Scartelaos* to form a cluster. Our data indicate that the kinship between *Periophthalmodon* and *Periophthalmus* is closer than those between *Periophthalmodon* and other selected genera.

**Figure 1.**
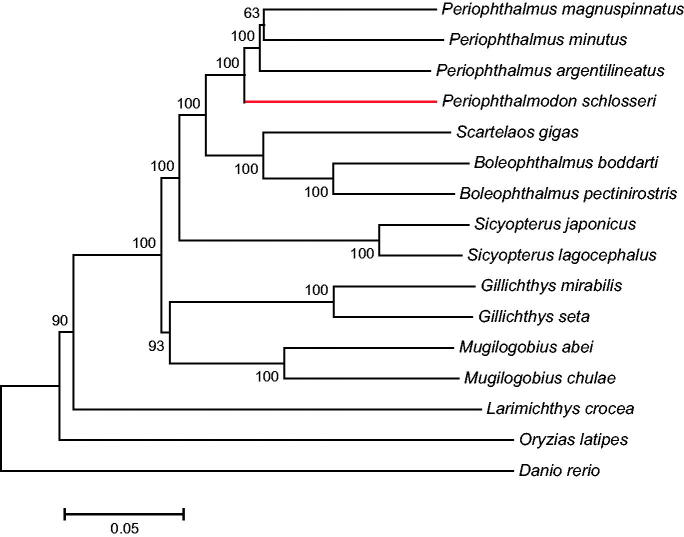
The phylogenetic tree (neighbour-joining topology) based on the comparison of whole mitochondrial genome sequences of 16 species. Numbers at each node represent the bootstrap value for neighbour-joining analysis. The nucleotide sequences from *P. magnuspinnatus* (KT284931.1), *P. minutus* (LK391944.1), *P. argentilineatus* (KT821095.1), *S. gigas* (KT277705.1), *B. boddarti* (KF874277.1), *B. pectinirostris* (JN631352.1), *S. japonicus* (JX628620.1), *S. lagocephalus* (KF482068.1), *G. mirabilis* (FJ211845.1), *G. seta* (FJ211846.1), *M. abei* (KF128984.1), *M. chulae* (KP144793.1), *L. crocea* (EU339149.1), *O. latipes* (AP004421.1), *D. rerio* (KM244705.1) were employed to construct the phylogenetic tree using MEGA6.06 software.
